# The risk of outpatient mental health care service use following departure from work: a cohort register study of migrant and non-migrant women

**DOI:** 10.1186/s12913-022-08113-z

**Published:** 2022-05-26

**Authors:** Melanie Straiton, Kamila Angelika Hynek, Karina Corbett

**Affiliations:** Department of Mental Health and Suicide, Norwegian Institute of Public Health, PO Box 222, 0213 Skøyen, Oslo Norway

**Keywords:** Migrants, Women, Mental disorder, Employment, Health service use

## Abstract

**Background:**

Non-workforce participation is associated with increased risk of mental disorder in the general population. Migrant women face disadvantage in the labour market but use mental health services to a lesser extent. This study investigates the risk of using mental health services following departure from the workforce among women in Norway, and if the strength of the relationship varies for migrant and non-migrant women.

**Methods:**

Using linked registry data, we followed a cohort of 746,635 women who had a stable workforce attachment over a three-year period. We used Cox proportional hazard models to determine the risk of using outpatient mental health services (OPMH) following departure from the workforce. We included an interaction analysis to determine if the relationship differed by migrant group and length of stay and conducted subsequent stratified analyses.

**Results:**

Departure from the workforce was associated with a 40% increased risk of using OPMH services among all women. Interaction analyses and subsequent stratified analyses indicated that departure from the workforce was associated with an increased risk of using OPMH services among non-migrant women and among women from countries outside of the European Economic Area, regardless of length of stay. For women from the European Economic Area with 2–6 years or 7–15 years in Norway, however, there was no increased risk.

**Conclusions:**

Departure from the workforce is associated with increased risk of mental health service use, also among migrant women. Migrant women as a group, are more often temporarily employed and therefore at greater risk of falling out of the workforce and developing a mental disorder. However, women with shorter length of stays may experience greater barriers to care and service use may be a poorer indicator of actual mental disorder.

**Supplementary Information:**

The online version contains supplementary material available at 10.1186/s12913-022-08113-z.

## Background

Migrants are at greater risk of mental health problems than the general population [1] and migrant women report higher levels of mental health problems than migrant men [2]. A recent study indicated that inequalities in workforce participation explain around 15% of mental health differences between migrants and non-migrants [3], suggesting that employment is important for mental health. In Europe, migrants from countries outside the European Union (EU) face disadvantage in the labour market and are less likely to be employed than the non-migrant population [4]. The difference in employment rates is larger for women than men. A similar pattern is observable in Norway, a member of the European Economic Area (EEA) [5]. Further, migrant women who are employed, are more likely to be in temporary employment [6] and thus have a greater risk of work departure than non-migrant women.

In the general population, non-workforce participation is associated with poorer mental health [7], increased risk of mental disorders [8] and use of mental healthcare services [9]. There may be a causal association; that workforce participation has a positive impact on mental health due to both material and psychological gains [10] while departure from the workforce results in loss of resources, status and reduced well-being. There is also a selection effect; those with mental disorders are less likely to (re-)enter or stay in the workforce [11–13]. Both explanations may be equally important for women [7].

Although cross-sectional studies indicate that workforce participation is also associated with better mental health among migrants [14–16], few studies with migrants have taken pre-existing differences into account. Previous history of mental disorder is important to consider because following the uptake of treatment for mental disorder, migrants are at greater risk of long-term unemployment than the non-migrant population [17]. A Swedish register study appears to be the only longitudinal study on departure from work and subsequent use of mental health services among migrants compared to the non-migrant population [18]. Overall, departure from work was associated with increased risk of hospitalisation for depressive disorder, but the relationship was stronger for migrant women than for migrant men, non-migrant men and non-migrant women. This study excluded individuals with a history of depressive disorder, thus accounting for some pre-existing differences in mental health.

Hospitalisations however, only account for a small proportion of mental disorders severe enough to require specialist treatment. Using outpatient mental health services as a proxy for mental disorder may give a better indication of the impact of departure from work on moderate to severe mental health disorders. Further, Hollander and colleagues did not consider differences across migrant groups [18]. In Norway, migrant women from the EEA are more likely to have higher education, be in employment and be financially better off compared with migrant women from outside the EEA [5, 19, 20]. Whilst migrant women from European countries come to Norway primarily for work or family reunification, women from African and Asian countries come primarily for family reunification or protection [21]. Women from EEA countries and non-EEA countries may therefore differ in both their likelihood of finding and staying in employment, as well as their risk of mental disorder.

Length of stay may also play a role in the relationship between departure from work and risk of mental disorder. Migrant women with shorter stays may have less secure jobs and be simultaneously entitled to fewer social welfare benefits, increasing the risk of mental disorder. Yet, migrant women with shorter stays are less likely to use outpatient care [22]. Knowing more about differences in risk of mental health service use upon departure from work by region of origin and length of stay may be important for planning interventions that promote mental health among migrant women and for health service planning in times of sudden economic downturn, such as during a pandemic.

The aim of the study was two-fold; 1) to determine if departure from work is associated with increased risk of using outpatient mental health (OPMH) services among stably employed migrant and non-migrant women living in Norway and 2) to see if the association between workforce participation status and OPMH service use differs for migrant women from EEA countries and other countries (non-EEA) with different lengths of stay compared to non-migrant women.

## Methods

### Study design

This was a cohort study design where baseline sample was selected out in 2009/2010 and followed from the 1^st^ January 2011 to the 31^st^ December 2013. The exposure, workforce participation, was time-dependent. STROBE guidelines were adhered to [23]. The research was conducted in accordance with the Declaration of Helsinki and approved by the Regional Committee for Medical and Health Research Ethics, South East Norway (REK 2014/1970).

### Data source

Several national registries/databases were linked at an individual level, using a de-identifiable version of the unique personal number assigned to all Norwegian-born individuals at birth and to residents with a stay of six or more months. The National Database for the Reimbursement of Health Expenses contains information on all patient contacts with OPMH services. The National Population Register contains demographic data of all residents in Norway and the National Education Database contains information on educational attainment and enrolment in current studies. Information on income was extracted from the National Income Registry and employment histories were extracted from the FD-Trygd database.

### Study population

The study population included women aged 26–62 who resided in Norway as of January 1^st^ 2011, and who, in the preceding two years (2009 and 2010) were employed and living in Norway. This age range was chosen since younger women may be more likely to still be in, or be planning to enter, higher education. Employment was defined as having earned 1.5 times the threshold for taxation in both 2009 and 2010 and not being enrolled in education in either year. This threshold has previously been used as an indicator of stable labour market attachment and is adjusted yearly for expected salary growth [24]. In 2021, 1.5 times the basic threshold for taxation was 159,599 Norwegian crowns (currently around 16,300 euros) [25]. Women were followed from the 1^st^ January 2011 until the date of their first OPMH appointment or were censored upon death, emigration, enrolment in education or the end of the follow-up period (31^st^ December 2013).

### Variables

Exposure: Workforce participation was based on employment entry and exit dates registered in FD-Trygd. A woman was considered employed starting the date she entered employment, and out of the workforce on the date her employment period ended. Since this variable was time dependent, she could move back and forth between these categories every time her employment status changed.

Outcome: Contact with OPMH services during follow-up period (no/yes).

Migrant group: Migrant women, defined as women born abroad with two foreign born parents, were divided into two groups based on country of origin: A country in the European Economic Area (EEA migrant women) or a country outside of the European Economic Area (non-EEA migrant women). All other women, those born in Norway or born abroad with at least one Norwegian-born parent, were classed as non-migrant women.

#### Covariates measured at the start of follow up

Length of stay: Calculated on a yearly basis: 2–6 years, 7–15 years, 16 + years. This grouping was chosen to represent short, middle and long length of stays. OPMH service use appears to be lower with shorter length of stays and flattens out after around 15 years [22].

Age-group: Calculated on a yearly basis: 26–36, 37–47 and 48–62 years.

Educational attainment: Higher, secondary, < secondary/unknown. Some migrants do not convert qualifications upon arrival. Their use of OPMH is similar to those with < secondary education and so they were combined together.

Civil status: Married, never married, previously married (separated, divorced or widowed).

Income: Low, middle, high. Low and high were defined as 20% under and over the median respectively.

Previous history of OPMH: Women who had had at least one OPMH consultation in 2009 or 2010 were classed as having a history of OPMH use.

### Statistical analysis

We calculated the incidence rates of first OPMH service use per 1000-person-years by workforce participation, migrant group and length of stay. For migrant women, we then combined length of stay and migrant group into one variable and calculated incidence rates and fitted Cox proportional hazard models to determine the relationship between workforce participation and time to first OPMH service use for all women. First, we only included the combined variable migrant group/length of stay variable and workforce participation, then we adjusted for previous OPMH use. It is important to control for confounding effects of, rather than exclude those with, previous history of OPMH service use since researchers have argued this can lead to bias in the findings [26]. Next, we added birth cohort, civil status, education and income level at baseline before introducing an interaction term between workforce participation and migrant group/length of stay. Finally, we conducted two sensitivity analyses; one where we excluded women who had a history of OPMH use and one where we excluded women who gave birth during the follow-up period. This was because mothers who take a temporary break from the workforce beyond standard parental leave may represent a different group than women who otherwise voluntarily or involuntarily leave the workforce (which may more often be due to unemployment or health-related reasons, both of which are likely to have a stronger association with mental disorder). We analysed the data in STATA version 17.

## Results

### Sample population

Our source population consisted of 1,191,946 women aged 26–62 residing in Norway as of 1^st^ January 2011. We excluded women who were not resident in 2009 or 2010 (*n* = 48 565) and who did not meet employment criteria (studying or earning less than 1.5 times the basic taxation amount) (*n* = 396,746). Our final sample population included 746,635 women who contributed 2,014,109 person years to the study. Table 1 gives an overview of the baseline characteristics of the sample by migrant group.

**Table 1:** Baseline demographic variables by migrant group

	**Non-migrant women**	**EEA migrant women**	**non-EEA migrant women**
Total N	681 159	28 374	37 102
Age group			
26–36 years	172 896 (25.38%)	10 453 (36.84%)	13 791 (37.17%)
37–47 years	234 107 (34.37%)	9621 (33.91%)	14 512 (39.11%)
48–62 years	274 156 (40.25%)	8300 (29.25%)	8799 (23.72%)
Civil status			
Never married	205 276 (30.14%)	8400 (29.60%)	3599 (9.70%)
Married	365 478 (53.66%)	15 725 (55.42%)	26 048 (70.21%)
Previously married	110 405 (16.21%)	4249 (14.97%)	7455 (20.09%)
Education			
< secondary/unknown	187 183 (27.48%)	3959 (13.95%)	12 027 (32.42%)
Secondary	197 005 (28.92%)	6185 (21.80%)	8701 (23.45%)
Higher	296 971 (43.60%)	18 230 (64.25%)	16 374 (44.13%)
Income			
Low	166 608 (24.46%)	8117 (28.61%)	14 226 (38.34%)
Middle	322 699 (47.37%)	11 579 (40.81%)	16 328 (44.01%)
High	191 852 (28.17%)	8678 (30.58%)	6548 (17.65%)
Length of stay			
2–6 years		8996 (31.71%)	5867 (15.81%)
7–15 years		8798 (31.01%)	14 644 (39.47%)
16 + years		10 610 (37.39%)	16 591 (44.72%)
History of OPMH use	19 700 (2.89%)	818 (2.88%)	1122 (3.02%)

### Analyses

The overall incidence of OPMH was 1.48 (95% CI = 1.46–1.50) per 1000 person-years. Table 2 shows person-years at risk, the overall OPMH incidence rates and number of cases by workforce participation, migrant group and length of stay separately. The incidence was higher for those who were out of the workforce compared to those who were employed. The incidence rate was also higher for both migrant groups than for non-migrant women. Migrant women who had lived in Norway for 7–15 years and 16 + years at baseline had a higher incidence of OPMH service use than non-migrant women, while it was not significantly different for migrant women with 2–6 years in Norway.

**Table 2:** Time at risk, OPMH use incidence rate per 1000-person-years and number of OPMH cases

	**Time at risk in years**	**Incidence rate per 1000 person years (95% CI)**	**Total OPMH cases**
By workforce participation			
Employed	692 731 949	1.39 (1.37–1.41)	26 396
Out of workforce	42 921 224	2.92 (2.83–3.02)	3434
By migrant group			
Non-migrant women	1 843 530	1.45 (1.44–1.47)	26 818
EEA women	74 063	1.71 (1.62–1.62)	1266
non-EEA women	96 516	1.81 (1.73–1.90)	1746
By length of stay			
2–6 years	37 273	1.50 (1.38–1.63)	560
7–15 years	60 558	1.91 (1.80–2.02)	1156
16 + years	72 747	1.78 (1.69–1.88)	1296

**Fig. 1:**
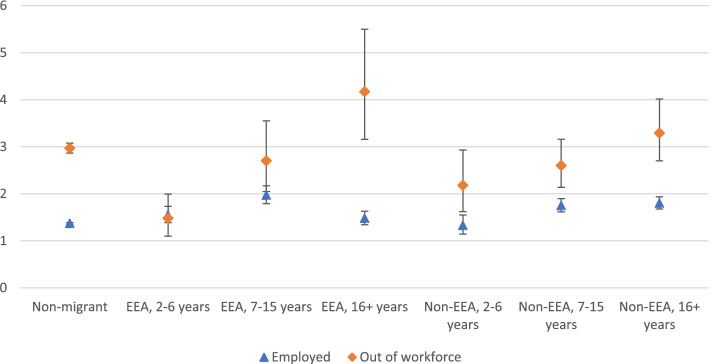
OPMH use incidence rate (95% CIs) per 1000-person-years

Since the OPMH rates differed across both migrant groups and length of stay, we combined migrant group and length of stay for migrants into one variable to see if the relationship between workforce participation and OPMH by length of stay was similar for both migrant groups in the main analyses. Figure 1 displays OPMH incidence rates per 1000 person years by migrant group, length of stay and workforce participation. Incidence rates for EEA women with 15 years or less in Norway were similar for the employed and those out of the workforce, while the incidence was almost three times higher for EEA women who were out of the workforce compared to employed EEA women with more than 15 years in Norway. For non-EEA women, incidence rates were higher for women out of the workforce than among employed, regardless of length of stay.

Table 3 displays the risk of OPMH by workforce participation status, migrant group and length of stay. Women who departed the workforce were at higher risk of OPMH use. Model 1 shows the unadjusted hazard ratios (HR). Model 2 is adjusted for history of OPMH service use. Inclusion of this variable explained some of the relationship between departure from work and OPMH. In model 3, where we adjusted for all significant covariates (age, civil status, education and income level), departing the workforce increased the risk of OPMH service use by 40% compared with employed women. EEA women with stays over 7 years and non-EEA women with stays over 15 years had a higher risk of service use compared to their non-migrant counterparts.

**Table 3:** Hazard ratios for OPMH service use by workforce participation and migrant group/length of stay

	**Model 1**	**Model 2**	**Model 3**	**Model 4**
Employed	1.00	1.00	1.00	1.00
Out of workforce	2.00 (1.93–2.08)***	1.65 (1.59–1.71)***	1.40 (1.35–1.45)***	1.43 (1.38–1.49)***
Non-migrant	1.00	1.00	1.00	1.00
EEA, 2–6 years	0.97 (0.88–1.08)	1.13 (1.01–1.25)*	0.94 (0.84–1.05)	1.03 (0.92–1.15)
EEA, 7–15 years	1.34 (1.23–1.47)***	1.30 (1.18–1.42)***	1.20 (1.09–1.31)***	1.24 (1.13–1.37)***
EEA, 16 + years	1.11 (1.01–1.21)*	1.10 (0.99–1.20)	1.28 (1.16–1.40)***	1.24 (1.13–1.37)***
non-EEA, 2–6 years	0.90 (0.79–1.03)	1.02 (0.88–1.16)	0.88 (0.77–1.01)	0.91 (0.78–1.06)
non-EEA, 7–15 years	1.19 (1.11–1.29)***	1.19 (1.10–1.28)***	1.04 (0.97–1.13)	1.07 (0.99–1.17)
non-EEA, 16 + years	1.29 (1.21–1.38)***	1.22 (1.14–1.31)***	1.25 (1.17–1.34)***	1.27 (1.18–1.37)***
Out of workforce*EEA, 2–6 years				0.57 (0.41–0.78)***
Out of workforce*EEA, 7–15 years				0.72 (0.54–0.97)*
Out of workforce*EEA, 16 + years				1.32 (0.98–1.78)
Out of workforce*non-EEA, 2–6 years				0.87 (0.62–1.21)
Out of workforce*non-EEA, 7–15 years				0.84 (0.68–1.04)
Out of workforce*non-EEA, 16 + years				0.86 (0.70–1.07)

Model 1: unadjusted analyses, Model 2: adjusted for OPMH history, Model 3: Adjusted for OPMH history, age group, income level, civil status, and education level. Model 4: Interaction analyses adjusted for OPMH history, age group, income level, civil status, and education level

*p*-values: *** < 0.001; ** < 0.01, * < 0.05

In model 4, we included an interaction term between migrant group by length of stay and workforce participation. The main effect of workforce participation on OPMH was significant (HR = 1.43, 95% CI = 1.38–1.49), indicating that departure from work was associated with an increased risk of OPMH service use among non-migrant women. The interaction term was significant for EEA women with 2–6 years and 7–15 years in Norway at baseline, and the hazard ratio suggested that the relationship between workforce participation and OPMH use was significantly weaker for these two groups of women.

Given the significant interaction, we stratified the analyses by migrant group and length of stay to better understand relationship between workforce participation and OPMH use for each group. Table 4 shows the fully adjusted hazard ratios for OPMH use by workforce participation, stratified by migrant group/length of stay. Departure from work was not associated with increased risk of OPMH for EEA migrant women with 2–6 years or 7–15 years in Norway at baseline. The HR for non-EEA migrant women with 2–6 years in Norway was marginally significant, although the estimate suggested a 35% higher risk. This lack of significance is due to the relatively small number of women in this group who were both out of the workforce and used OPMH services (*N* = 44). For all other groups, departure from work was associated with significantly higher risk of service use.

**Table 4:** Hazard ratio for OPMH service use by workforce participation^a^: Stratified by group

	**Non-migrant**	**EEA, 2–6 years**	**EEA, 7–15 years**	**EEA, 16 + years**	**non-EEA, 2–6 years**	**non-EEA, 7–15 years**	**non-EEA, 15 + years**
Employed	1.00	1.00	1.00	1.00	1.00	1.00	1.00
Out of the workforce	1.42 (1.36–1.47)***	0.86 (0.62–1.19)	1.10 (0.82–1.48)	1.98 (1.46–2.68)***	1.35 (0.96–1.90)^	1.27 (1.03–1.58)*	1.34 (1.08–1.67)**

^a^adjusted for history of OPMH service use, age-group, civil status, education and income level; *p*-values: *** < 0.001; ** < 0.01, * < 0.05; ^ < 0.10. *EEA* European Economic Area, *OPMH* Outpatient mental healthcare

We then repeated the analyses, first excluding all women who gave birth during the follow up period (*N* = 62 204). See additional file 1 for the main model, interaction analyses and stratified analyses. Secondly, we excluded all women with a history of OPMH service use (*N* = 21 640). See additional file 2. We found similar results in both sensitivity analyses, demonstrating the robustness of the findings.

## Discussion

The aim of this study was to look at the relationship between departure from work and risk of using outpatient mental health services. In line with other studies, the overall risk of using services was higher for women who fell out of the workforce than women who were employed [9, 18]. Notably, departing work was associated with greater risk of OPMH use among non-migrant women, migrant women from non-EEA countries and migrant women from EEA countries with more than 15 years in Norway. It was not however, associated with higher risk of OPMH use among EEA migrant women with less than 15 years. This could be because EEA women are often labour migrants and due to freedom of movement within the EEA, many EEA migrants emigrate from Norway [27]. Labour migrants with shorter stays are more likely to return to their home country than migrants who have settled in a country for longer [28]. As a result, EEA migrant women may be returning home before we observe them falling out of the workforce or before the onset of a mental disorder and use of OPMH services. In contrast, a higher proportion of migrant women from non-EEA countries come to Norway as refugees or to reunite with family and are therefore less likely to return to their home countries following job loss. It is also possible that EEA migrant women seek treatment in their home country while living in Norway. Service use in the home country appears to decline with increasing length of stays in the new country [29].After 15 years, EEA migrants may be permanently settled and more inclined to use health services in Norway. They may also have more financial obligations and job loss could have greater mental health consequences. Thus, the relationship between OPMH use and workforce participation becomes more apparent.

A previous study found that the relationship between departure from work and hospital admittance for depression was stronger for migrant women than non-migrant women in Sweden [18]. This is in contrast to our study where the relationship was not significantly stronger for any group of migrant women than for non-migrant women and does not support the theory that the most disadvantaged groups experience the greatest mental health consequences of falling out of the workforce [30]. The difference in findings could be due to the more specific outcome measure in the Swedish study, or our looser definition of previous attachment to the workforce. Hollander and colleagues only included women who were employed full-time, while we included women regardless of how many hours they worked. Departure from a full-time job could be more financially and psychosocially devastating for migrant women than departure from a part-time job relative to their non-migrant counterparts.

Interestingly, the overall differences in OPMH use between non-migrant and migrant women were relatively small in this study in contrast to an earlier study including migrant women [22]. This could be due to the exclusion of women who were not active in the labour market in the two years prior to inclusion in the current study. This could indirectly suggest that migrant women who have been employed experience fewer barriers to service use than women who have not participated in the workforce. Employed migrant women are presumably more active in society, have greater Norwegian proficiency, education and possibly better health literacy. These factors may be linked to more timely and appropriate health-care seeking [31, 32]. Thus, strategies to improve identification of mental disorder and treatment seeking among migrant women should be directed at those who do not participate in the workforce.

A major limitation of this study is that women who depart the workforce may have done so voluntarily or involuntarily. Further, in the sensitivity analysis, exclusion of women who may voluntarily leave the workforce to bring up young children yielded strikingly similar results, suggesting that departure from work, regardless of reason, is associated with increased risk of OPMH use. Another limitation is that although we have attempted to ensure that the departure from work happens prior to OPMH use during follow-up and controlled for possible confounding of previous OPMH use, we cannot infer causation. It is possible that those with mental disorder have an increased risk of departure from work in the period between disorder onset and OPMH consultation. Further, since our sample included only women who had been in the workforce at least two years prior to inclusion, there may have been a healthy worker bias present, since those with a pre-existing mental disorder are less likely to be employed and therefore will have initially been selected out of the study sample [17].It should also be highlighted that among both EEA and non-EEA migrant women, newly arrived women have the lowest risk of service use. Yet, they also experienced more time out of the workforce (not shown). It is possible that newly arrived women have better mental health, regardless of employment status and that the risk of disorder increases over time. At the same time, women with shorter stays may also experience greater barriers to care [29]. Thus, use of mental health services is likely to be a poorer reflection of mental disorder for migrant women with shorter stays compared to women with longer stays. Future research should therefore also consider other measures of mental disorder in order to better understand the relationship between departure from the workforce and mental disorder among migrants. Finally, our definition of migrants (foreign-born with two foreign-born parents) may not capture all women who have had migration-related experiences, or who experience discrimination in the labour market based on their migrant background. Future research should therefore also consider the relationship between workforce departure and mental disorder among descendants of migrants and individuals who have migrated to Norway with at least one Norwegian-born parent.

## Conclusion

Departure from work is associated with higher risk of OPMH service use among women in Norway, except for EEA women with shorter stays. However, since migrant women, in general, are more often precariously employed, they are, as a group, more likely to fall out of the workforce than non-migrant women, and therefore be at greater risk of mental disorder. Policies which discourage precarious employment and instead help to ensure permanent positions may be beneficial for migrant women’s mental health. Further, the smaller differences in risk of using OPMH services among migrant and non-migrant women in this study compared to previous research might suggest that migrant women who have been active in the workforce experience fewer barriers to healthcare seeking than migrant women who have not been active. Workforce participation may therefore be a key factor for accessing and using mental health care services when needed. Thus, policies that encourage migrant women’s active participation in the workforce may also improve timely and appropriate health care use for mental disorder.

## Supplementary Information

**Additional file 1: **Analyses excluding women who gave birth during the follow-up period.

**Additional file 2:** Analyses excluding women with a history of outpatient mental health service use.

## Data Availability

The datasets generated and analysed for the current study are not publicly available due to data protection, but the data that support the findings of this study may be available from Statistics Norway and HELFO upon request and with appropriate ethical approval.

## Abbreviations

OPMH: Outpatient mental health
EU: European Union
EEA: European Economic Area
HR: Hazard ratio
