# Supplementary material for: The risk of outpatient mental health care service use following departure from work: a cohort register study of migrant and non-migrant women
Source: BMC Health Serv Res. 2022 May 26;22:706. doi: 10.1186/s12913-022-08113-z (PMC9137189; doi:10.1186/s12913-022-08113-z)
Supplement: Supplementary file 1 — Additional file 1: Analyses excluding women who gave birth during the follow-up period. [file 12913_2022_8113_MOESM1_ESM.docx]

**Additional files 1: Analyses excluding women who gave birth during the follow-up period**

Table 1 shows the hazard ratio for outpatient mental healthcare (OPMH) service use by workforce participation and migrant group/length of stay for women who did not give birth during the follow-up period (n=661 459). In model 3, the fully adjusted model, exiting the workforce was associated with a 53% increased in risk of OPMH use. According to the interaction analyses, model 4, the relationship between workforce participation and OPMH service use was significantly weaker for European Economic Area (EEA) migrant women with 2-6 years in Norway compared with non-migrant women and marginally weaker for EEA migrant women with 7-15 years in Norway. This is similar to the findings in the main analyses where a significant difference was found for both these groups compared to non-migrant women. In this model however, the relationship was also marginally weaker for non-EEA migrant women with 7-15 years in Norway compared with non-migrant women. In stratified analyses (table 2), exiting the workforce was still associated with a substantial, but not significant, increase in risk of OPMH use among non-EEA migrant women with 2-6 years in Norway (HR=1.36, 95%CI 0.91-2.03). As in the original analyses, exiting the workforce was not associated with an increased risk of mental disorder among EEA migrant women with 2-6 years and 7-15 years in Norway.

| **Table 1: Hazard ratios for OPMH service use by workforce participation and migrant group/length of stay^1^** | | | | |
| --- | --- | --- | --- | --- |
|  | **Model 1** | **Model 2** | **Model 3** | **Model 4** |
| Out of workforce | 2.35 (2.26-2.44)*** | 1.82 (1.75-1.89)*** | 1.53 (1.47-1.59)*** | 1.57 (1.51-1.64)*** |
| Non-migrant | 1.00 | 1.00 | 1.00 | 1.00 |
| EEA, 2-6 years | 1.08 (0.96-1.21) | 1.22 (1.09-1.37)*** | 0.95 (0.84-1.07) | 1.04 (0.92-1.17) |
| EEA, 7-15 years | 1.37 (1.25-1.52)*** | 1.32 (1.20-1.46)*** | 1.17 (1.06-1.29)** | 1.21 (1.09-1.34)*** |
| EEA, 16+ years | 1.08 (0.98-1.19) | 1.07 (0.98-1.18) | 1.27 (1.15-1.39)*** | 1.24 (1.12-1.37)*** |
| non-EEA, 2-6 years | 0.92 (0.79-1.07) | 1.04 (0.89-1.20) | 0.86 (0.74-0.999)* | 0.90 (0.76-1.06) |
| non-EEA, 7-15 years | 1.22 (1.12-1.32)*** | 1.21 (1.12-1.31)*** | 1.03 (0.96-1.12) | 1.07 (0.98-1.16) |
| non-EEA, 16+ years | 1.26 (1.17-1.35)*** | 1.19 (1.11-1.28)*** | 1.22 (1.14-1.31)*** | 1.25 (1.16-1.34)*** |
| Out of workforce*EEA, 2-6 years | |  |  | 0.56 (0.39-0.80)*** |
| Out of workforce*EEA, 7-15 years | |  |  | 0.73 (0.52-1.00)^ |
| Out of workforce*EEA, 16+ years | |  |  | 1.28 (0.94-1.73) |
| Out of workforce*non-EEA, 2-6 years | |  |  | 0.79 (0.54-1.16) |
| Out of workforce*non-EEA, 7-15 years | |  |  | 0.79 (0.63-1.00)^ |
| Out of workforce*non-EEA, 16+ years | |  |  | 0.84 (0.66-1.05) |
| ^1^Model 1: unadjusted analyses, Model 2: adjusted for OPMH history, Model 3: Adjusted for OPMH history, age group, income level, civil status, and education level. Model 4: Interaction analyses adjusted for OPMH history, age group, income level, civil status, and education level. ^p<0.10, *p<0.05, **p<0.01, ***p<0.001. EEA – European Economic Area; OPMH – Outpatient mental healthcare | | | | |

| **Table 2: Hazard ratio for OPMH service use by workforce participation^1^: Stratified by group** | | | | | | | |
| --- | --- | --- | --- | --- | --- | --- | --- |
|  | **Non-migrant** | **EEA, 2-6 years** | **EEA, 7-15 years** | **EEA, 16+ years** | **non-EEA,**  **2-6 years** | **non-EEA,**  **7-15 years** | **non-EEA,**  **15+ years** |
| Employed | 1.00 | 1.00 | 1.00 | 1.00 | 1.00 | 1.00 | 1.00 |
| Out of the workforce | 1.56 (1.49-1.62)*** | 0.95 (0.66-1.36) | 1.18 (0.85-1.65) | 2.10 (1.54-2.85)*** | 1.36 (0.91-2.03) | 1.34 (1.07-1.70)* | 1.52 (1.20-1.92)** |

**^1^** adjusted for age group, income level, civil status, and education level. ^p<0.10, *p<0.05, **p<0.01, ***p<0.001. EEA – European Economic Area; OPMH – Outpatient mental healthcare
