# Supplementary material for: The risk of outpatient mental health care service use following departure from work: a cohort register study of migrant and non-migrant women
Source: BMC Health Serv Res. 2022 May 26;22:706. doi: 10.1186/s12913-022-08113-z (PMC9137189; doi:10.1186/s12913-022-08113-z)
Supplement: Supplementary file 2 — Additional file 2: Analyses excluding women with a history of outpatient mental health service use. [file 12913_2022_8113_MOESM2_ESM.docx]

**Additional file 2: Analyses excluding women with a history of outpatient mental health service use**

Table 1 shows the hazard ratio for outpatient mental healthcare (OPMH) service use by workforce participation and migrant group/length of stay for women with no history of OPMH service use (n=724 995). In model 2, the fully adjusted model, falling out of the workforce is associated with a 47% increased risk of OPMH use. According to the interaction analyses, model 3, the relationship between workforce participation and OPMH service use was significantly weaker for European Economic Area (EEA) migrant women with 2-6 years and 7-15 years in Norway compared with non-migrant women. This is similar to the findings in the main analyses where a significant difference was found for both these groups compared to non-migrant women. In stratified analyses (table 2), leaving the workforce was associated with a significant increase in risk of OPMH use among all groups except for EEA migrant women with 2-6 years and 7-15 years in Norway. This is the same as demonstrated in the original analyses and therefore indicates the robustness of the findings.

| **Table 1: Hazard ratios for OPMH service use by workforce participation and migrant group/length of stay^1^** | | | |
| --- | --- | --- | --- |
|  | **Model 1** | **Model 2** | **Model 3** |
| Out of workforce | 1.85 (1.76-1.93)*** | 1.47 (1.40-1.54)*** | 1.51 (1.44-1.59)*** |
| Non-migrant | 1.00 | 1.00 | 1.00 |
| EEA, 2-6 years | 1.07 (0.95-1.22) | 0.83 (0.73-0.94)** | 0.93 (0.82-1.06) |
| EEA, 7-15 years | 1.42 (0.95-1.22)*** | 1.24 (1.11-1.38)*** | 1.30 (1.16-1.46)*** |
| EEA, 16+ years | 1.11 (0.99-1.25) | 1.34 (1.20-1.51)*** | 1.33 (1.18-1.50)*** |
| non-EEA, 2-6 years | 1.07 (0.92-1.25) | 0.86 (0.73-0.999)* | 0.86 (0.72-1.02) |
| non-EEA, 7-15 years | 1.23 (1.12-1.35)*** | 0.99 (0.90-1.08) | 1.02 (0.92-1.12) |
| non-EEA, 16+ years | 1.40 (1.29-1.52)*** | 1.38 (1.28-1.50)*** | 1.41 (1.30-1.54)*** |
| Out of workforce*EEA, 2-6 years | |  | 0.44 (0.29-0.67)*** |
| Out of workforce*EEA, 7-15 years | |  | 0.63 (0.43-0.93)*** |
| Out of workforce*EEA, 16+ years | |  | 1.14 (0.76-1.71) |
| Out of workforce*non-EEA, 2-6 years | |  | 0.96 (0.65-1.40) |
| Out of workforce*non-EEA, 7-15 years | |  | 0.82 (0.63-1.07) |
| Out of workforce*non-EEA, 16+ years | |  | 0.83 (0.63-1.08) |
| ^1^Model 1: unadjusted analyses, Model 2: Adjusted for age group, income level, civil status, and education level. Model 4: Interaction analyses adjusted for age group, income level, civil status, and education level. *p<0.05, **p<0.01, ***p<0.001. EEA – European Economic Area; OPMH – Outpatient mental healthcare | | | |

| **Table 2: Hazard ratio for OPMH service use by workforce participation^1^: Stratified by group** | | | | | | | |
| --- | --- | --- | --- | --- | --- | --- | --- |
|  | **Non-migrant** | **EEA, 2-6 years** | **EEA, 7-15 years** | **EEA, 16+ years** | **non-EEA, 2-6 years** | **non-EEA, 7-15 years** | **non-EEA, 16+ years** |
| Employed | 1.00 | 1.00 | 1.00 | 1.00 | 1.00 | 1.00 | 1.00 |
| Out of the workforce | 1.50 (1.43-1.58)*** | 0.71 (0.47-1.08) | 1.01 (0.68-1.48) | 1.72 (1.14-2.60)** | 1.60 (1.08-2.36)* | 1.35 (1.04-1.76)* | 1.28 (0.98-1.68)^ |

**^1^** adjusted for age group, income level, civil status, and education level. ^p<0.10, *p<0.05, **p<0.01, ***p<0.001. EEA – European Economic Area; OPMH – Outpatient mental healthcare
